# Investigation of the polymerization mechanism of ferrocene doped C_60_ under high pressure and high temperature

**DOI:** 10.1038/s41598-017-11425-4

**Published:** 2017-09-07

**Authors:** Shishuai Sun, Wen Cui, Shuangming Wang, Bingbing Liu

**Affiliations:** 1 College of science, Tianjin University of Technology, Tianjin, 300384 China; 2 College of Physics and Materials Science, Tianjin Normal University, Tianjin, 300387 China; 3 State Key Laboratory of Superhard Materials, Jilin University, Changchun, 130012 China

## Abstract

*In situ* high pressure and high temperature (HPHT) study has been carried out on C_60_/ferrocene (Fc) in order to detect the process of polymerization and reveal the polymerization mechanism. Pristine C_60_ was also studied under same conditions for comparison. In both cases, similar types of polymers can be observed after pressure and temperature release, but with different fractions, i.e. a larger amount of 2D polymers were formed in pure C_60_, while more branch-like polymers were synthesized in C_60_/Fc, although the most fraction of the polymers is still 1D chain-like polymer in both of the materials. The polymers formed in C_60_ can be detected both during the “up” run (pressure and temperature increase) and the “down” run (pressure and temperature decrease), while in C_60_/Fc, the polymers can only be synthesized in the “down” run. The differences between the two cases were attributed to the different initial lattice structures of the two materials and the confinement effect of the dopant. The polymerization mechanism on C_60_/Fc under HPHT was also revealed in this work.

## Introduction

Doping process can greatly improve the electrical and optical features of materials, which are very important for high technical products^1–5^. Among these, doped fullerene materials have aroused a research fever for creation of new materials, by using molecular confinement or co-intercalation by template molecules before the reaction^4, 5^. More importantly, doping is effectively used to synthesize controllable pressure induced polymerized fullerene. For example, a reversible orthorhombic polymerization has been observed in Na_2_RbC_60_ and Na_2_CsC_60_ under pressure at room temperature^6^. Popov *et al*. reported that a 3D-polymerized C_60_ with high bulk module of 585 GPa was formed in C_60_/CS_2_ at pressure 6–7 GPa and room temperature^7^. The covalent bonds between C_60_ molecules were formed in {Cd(dedtc)_2_}_2_*C_60_ under pressure^8^. Moreover, pressure-induced metastable ordered polymers were synthesized from C_60_/AgNO_3_ and C_60_/Ni(OEP) under high pressure^9^. Both the quenched materials contain chain-like polymers and dimers but with different fractions, which can be understood by the different initial lattice structures of these materials and the confinement effects of the dopants. Besides polymerization, doped C_60_ materials and some other carbon materials can also transform into many novel phases with various properties under pressure. For example, long range ordered structures with the building block of amorphized C_60_ and C_70_ cluster were synthesized from C_60_/m-xylene and C_70_/m-xylene under high pressure, respectively, which phases were superhard and can indent diamond anvils^10–12^. Wang *et al*. synthesized several new crystal structures of carbon (e.g., bct-C4, H-, M-, R-, S-, W-, and Z-carbon) from compressed graphite^13, 14^. Therefore, applying pressure is a useful tool to synthesize new materials from doped C_60_ and carbon based materials, since pressure can tune the distance between neighboring molecules to form new bonds.

Besides, temperature is another important factor to obtain new C_60_ based materials. Combination of the two technical methods can bring us some new phenomena and obtain more new phases, especially unique polymeric structures, with novel properties^15–19^. For example, Yamanaka *et al*.^15^. Obtained a body-centered orthorhombic 3D C_60_ polymer from a 2D polymeric C_60_ single crystal at 15 GPa and 600 °C. In contrast with the nonconductive behavior of 2D polymers, the obtained 3D C_60_ polymer was electronically conductive. In our recent study, metastable 1D or 2D polymers were formed from C_60_/Fc, C_60_/AgNO_3_ and C_60_/Ni(OEP) after high pressure and high temperature (HPHT) treatment^20^. We found that due to the spatial and geometrical confinement effects of the dopants, the polymerization degree is always lower than that of pure C_60_ treated at the same conditions. Meanwhile, the polymeric phases formed in the three doped materials were also different, which was attributed to the unique initial lattice structures and the different degrees of spatial confinement provided by the dopants. However, in this HPHT method it is difficult to detect and study the phase transition process of the samples. The polymerization mechanism in doped C_60_ materials under HPHT is still unclear. Thus, searching a new method to track the process of polymerization is a challenge subject.

In this study, we used a self-assembly external heating assembled diamond anvil cell (DAC) to *in situ* study the polymerization of a typical doped C_60_ material, C_60_/Fc, up to 3.2 GPa and 190 °C. The usage and assembly details of the DAC were similar as that presented in previous works^21, 22^. To reveal the process of polymerization, pristine C_60_ was also treated under same conditions for comparison. Our results showed that both C_60_ and C_60_/Fc can form similar types of polymers under studied conditions but with different fractions. In C_60_ the polymers were formed under all of our studied conditions, while in C_60_/Fc, the polymers can only be synthesized when pressure and temperature release. We found that the different initial lattice structures of these materials and the confinement effect of the dopant were contributed to the differences between the two cases. The polymerization mechanism on C_60_/Fc was also uncovered in this study. This work also opens a new sight on the research method of phase transition in doped C_60_ materials and establishes favorable foundation on the synthesis of new materials with various phases and unique properties.

## Results and Discussion

### Structure of C60/Fc

**Figure 1:**
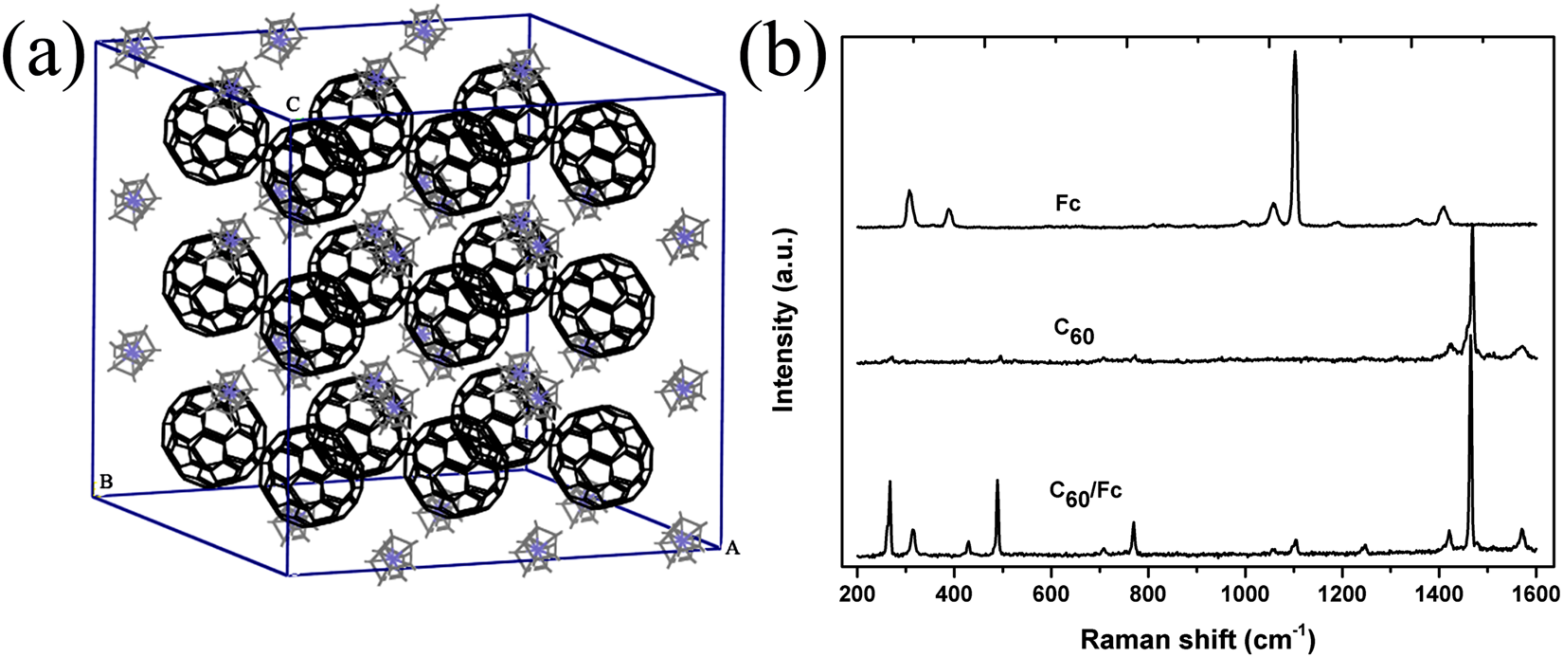
The packing arrangement of C_60_/Fc (**a**) (The hourglass-like shape stands for Fc and the ball-like shape represents for C_60_) and the Raman spectra of Fc, C_60_ and C_60_/Fc (**b**).

The packing arrangement of C_60_/Fc was shown in Fig. 1a (calculated by Materials Studio from the data in ref. 23). C_60_/Fc has a unique layered structure which consists of close-packed layers of C_60_ molecules stacked directly one above the other. To study the structure of C_60_/Fc, Raman spectroscopy was employed and the result was shown in Fig. 1b. The spectra of pure C_60_ and the dopants were also presented in Fig. 1b for comparison. The spectrum of pristine C_60_ contains ten peaks, including eight Hg modes and two Ag modes. From this figure, we can see that the spectrum of C_60_/Fc contains Raman signals from both the intercalant molecules and from C_60_, which indicates that the dopants are efficiently intercalated to the lattice of C_60_.

### Results of the samples treated after HPHT

**Figure 2:**
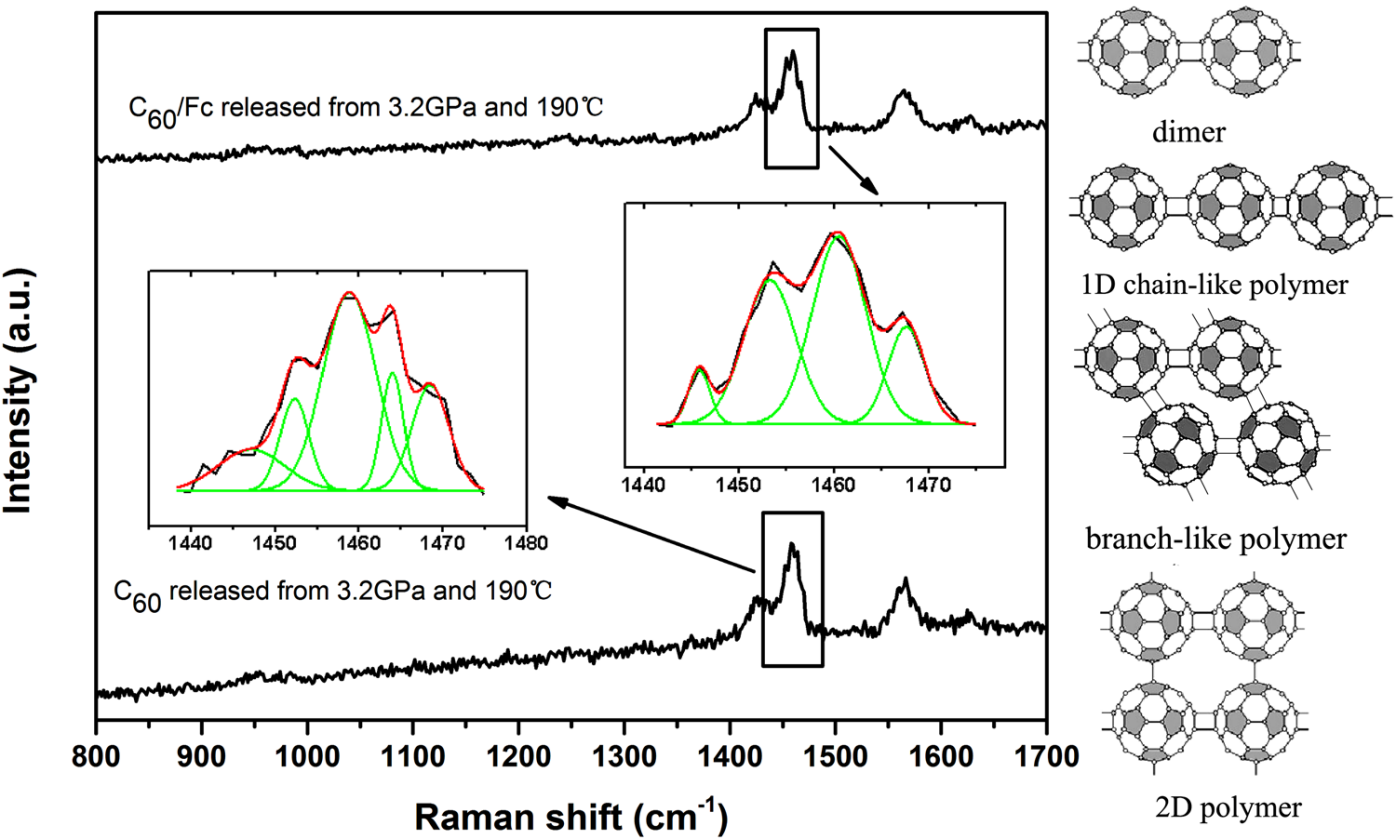
The Raman spectra of C_60_/Fc (top) and C_60_ (bottom) released from 3.2 GPa and 190 °C. The magnified Ag(2) modes were shown in the insets.

We first present the results of pure C_60_ and C_60_/Fc after the treatment of 3.2 GPa and 190 °C. The Raman spectra for the two samples were shown in Fig. 2. We also magnified the Ag(2) mode in the insets, since polymers are usually identified from the shift of the high-intensity Raman Ag(2) mode, which at room temperature falls at 1469 cm^−1^. The formation of intermolecular bonds shifts the electron density away from the remaining double bonds, which became weaker. This causes a shift of about 5 cm^−1^ for dimers, a shift of 10 cm^−1^ for linear chains, and 21 cm^−1^ for tetragonal phase which has been thoroughly studied and generally accepted in the field of fullerene studies^24–28^. From this figure, we can see that for pure C_60_, the Ag(2) peak splits into five peaks with positions at 1468, 1464, 1459, 1452 and 1447 cm^−1^ (shown in the left inset of Fig. 2), indicating that the quenched sample is a mixture of free molecules, dimers, 1D chain-like polymers, branch-like polymers and 2D polymers, respectively^24, 25^. For C_60_/Fc, the Ag(2) peak splits into four peaks with positions at 1467, 1460, 1453 and 1446 cm^−1^ (shown in the right inset of Fig. 2), which indicated that the quenched sample is a mixture of free molecules, 1D chain-like polymers, branch-like polymers and 2D polymers, respectively. Further analyzing, we found that although both of the quenched samples contained similar components of polymers, the fractions are very different, especially the fractions of branch-like polymers and 2D polymers, which can be easily detected by the areas of the peaks at around 1452 and 1447 cm^−1^, respectively. The areas of the peak at around 1447 cm^−1^ are 12.1% and 5.1% in quenched C_60_ and C_60_/Fc, respectively, and the percentages are 11.9% and 33.6% for the areas of the peak at around 1452 cm^−1^. This result demonstrated that a larger amount of 2D polymer was synthesized from pure C_60_, while more branch-like polymers were obtained from C_60_/Fc. It should be noted, however, the most fraction of the polymers is still 1D chain-like polymer in both of the two samples, due to the largest area of the peak at around 1460 cm^−1^.

The different fractions of the obtained polymers formed in quenched C_60_ and C_60_/Fc can be understood by the different initial lattice structures of the samples. Because pure C_60_ is free from steric effects, the polymerization is random, a large number of randomly oriented dimers can be obtained first and these can grow in any direction to form straight chains, branched chains, 2D polymers and possibly large amorphous structures. Meanwhile, the initial fcc structure makes it easily to form 1D chain-like polymers first and then 2D T-phase polymers (the basic polymeric structures were shown on the right of Fig. 2) in our studied conditions. For C_60_/Fc, the geometrical separation of the C_60_ layers prevents the formation of intercage polymer bonds in the c direction (see Fig. 1a). Thus, the polymerization in C_60_/Fc can be more likely to proceed within each fullerene layer. The special plane distribution of C_60_s makes it more possible to form 1D chain-like polymers first and then branch-like polymers under high temperature and high pressure, while only a few fraction of inter-layered C_60_s can form 2D square-like polymers (similar to the 2D T-phase polymers formed in pure C_60_). The most possible polymeric structures were also drawn in Fig. 2.

### *In situ* HPHT study on C_60_ and C_60_/Fc

**Figure 3:**
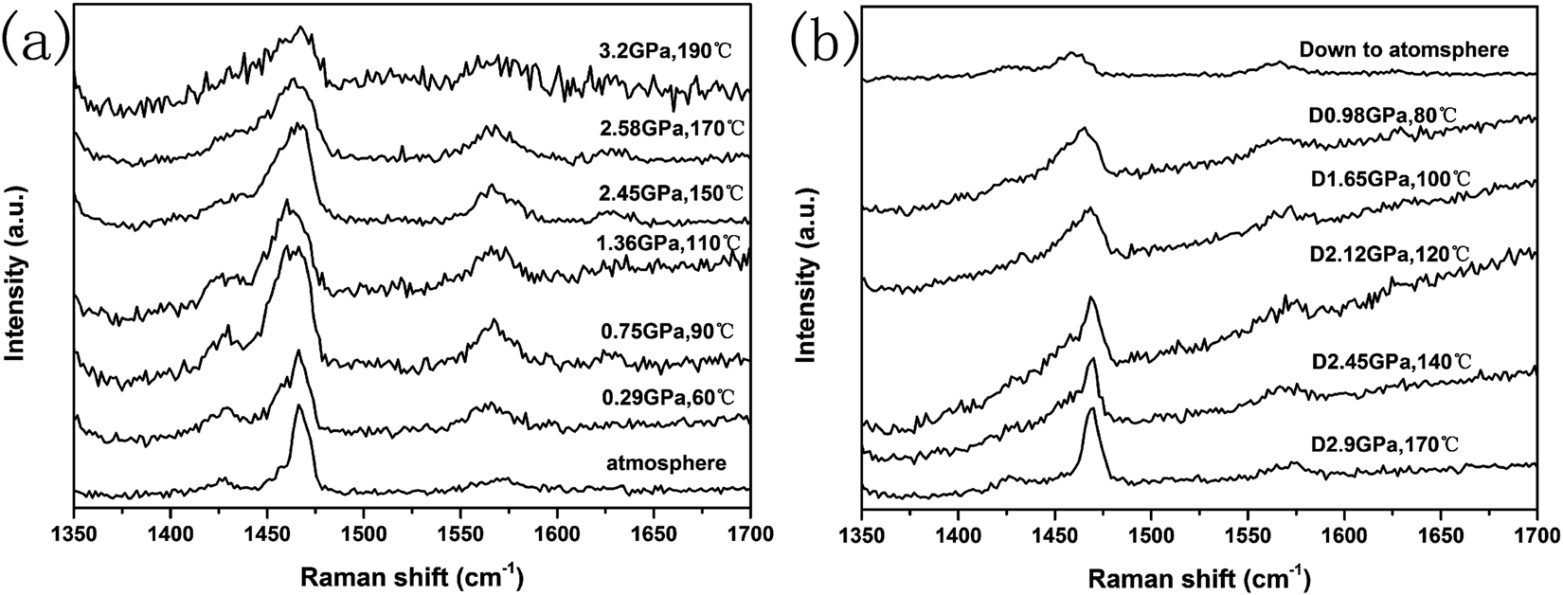
The Raman spectra of C_60_ recorded at different pressures and temperatures in the “up” run (**a**) and “down” run (**b**), respectively.

To further study the formation process of the polymers and reveal the polymerization mechanism on C_60_/Fc, *in situ* HPHT Raman study was carried out on C_60_ and C_60_/Fc. Figure 3a,b show the Raman spectra of C_60_ recorded at different pressures and temperatures in the “up” run and “down” run, respectively. From Fig. 3a, we can see that as the pressure and temperature increasing, the Ag(2) modes become broad and a wide shoulder centered around 1455 cm^−1^ at the lower frequency of Ag(2) was appeared from 0.29 GPa and 60 °C. The shoulder can still be observed up to 3.2 GPa and 190 °C. This indicated that C_60_ can form polymers from very low pressure and temperature and the polymers can always exist under the studied conditions. When the pressure and temperature gradually released to atmosphere condition, a broad shoulder at the lower frequency of Ag(2) was appeared from 2.45 GPa and 140 °C and the shoulder can be observed in all of the studied conditions. Until the pressure and temperature released to atmosphere, the Ag(2) mode obviously split to five peaks as we have mentioned above. These results indicated that the polymeric phases can be synthesized both in the “up” run and “down” run.

**Figure 4:**
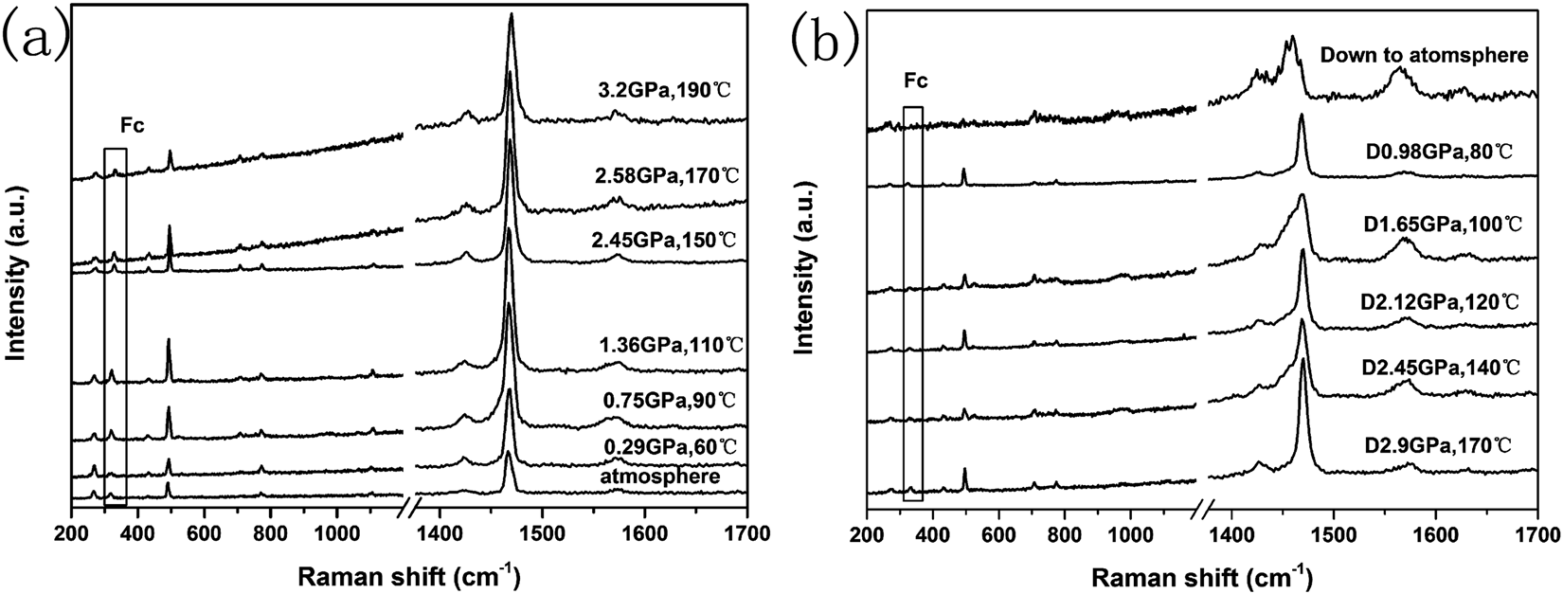
The Raman spectra of C_60_/Fc recorded at different pressures and temperatures in the “up” run (**a**) and “down” run (**b**), respectively. The modes from Fc were marked by rectangles.

The Raman spectra of C_60_/Fc under different pressures and temperatures were shown in Fig. 4. As the pressure and temperature increasing, it was found that the Ag(2) modes did not change a lot and the positions were always located at around 1467 cm^−1^ (shown in Fig. 4a), which was very different from the phenomenon observed in pure C_60_ under the same conditions. However, as the pressure and temperature decreasing, a broad shoulder at the lower frequency of Ag(2) was appeared from 2.45 GPa and 140 °C which is similar as the phenomenon observed in pure C_60_ and the shoulder can also be observed in most of the studied conditions. Until the pressure and temperature released to atmosphere, we found that the Ag(2) mode obviously split to four peaks as we have mentioned above. These results demonstrated that the polymers cannot be formed in the “up” run, but in the “down” run the polymers were gradually synthesized.

The different formation process of the polymers synthesized from C_60_ and C_60_/Fc can also be reasonably explained by their different initial lattice structures. Due to the existence of Fc molecules in C_60_/Fc, which can interact with the C_60_ molecules to affect the rotation and the available volume for the C_60_ molecules under HPHT, the formation of polymers is more difficult than that in pure C_60_ under the same conditions. Thus only at suitable pressures and temperatures can the polymers be formed in C_60_/Fc, while for pure C_60_, the free steric effects made it easily to form different polymers under various pressures and temperatures.

**Figure 5:**
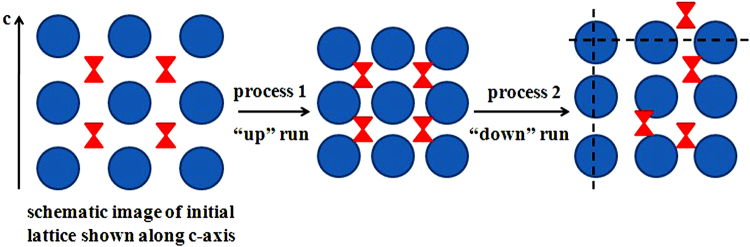
The schematic image of the polymerization mechanism. The blue solid circle represents C_60_ molecule and the red hourglass-like shape represents Fc molecule.

Further analyzing, we found that the peak positioned at around 317 cm^−1^ which represented the vibrational modes from the inserted Fc molecules (marked by rectangles in Fig. 4) could be always obviously observed up to the highest pressure and temperature in the “up” run. However, only very weak signals can be detected in the “down” run. We thus try to describe the polymerization mechanism as follows and draw the schematic image of this mechanism in Fig. 5 for clear understanding. In the “up” run (see process 1 in Fig. 5), although the Fc molecules can be gasified above 100 °C, the gaseous Fc molecules cannot leave the initial lattice but become more closed to neighboring molecules with the presence of pressure, which caused the effect of spatial confinement to hinder the bonding between C_60_s. Meanwhile, the temperature can affect the rotation of C_60_ molecules which makes the neighboring C_60_s may not face to each other in a favorable position for polymerization. That was the reason why no polymeric phases were obtained in the “up” run. However, in the “down” run (see process 2 in Fig. 5), as pressure decreasing the crystal lattice will slightly expand and the gaseous Fc molecules may move and depart from their original positions in a certain degree which induced some parts of C_60_ molecules can bond to each others to form polymers (the possible polymerized directions were drawn with imaginary line shown in Fig. 5), but some other parts of C_60_s are still separated by Fc molecules. That was the reason why only weak Raman signals of Fc can be observed in the polymeric phases shown in Fig. 4b and for some parts of quenched C_60_/Fc which cannot form polymers, the obvious peak of Fc molecules was still be observed (not shown in Fig. 4b).

The polymerization process of C_60_/Fc under HPHT is different from that of our previous study on C_60_/Fc under cold compression^29^, in which case that the 1D chain-like polymers were obtained when pressure increased to 5 GPa. However, higher dimensional polymers, such as 2D polymers, cannot be observed in that case. The differences verified that temperature plays an important role in the process of higher dimensional polymerization between neighboring C_60_ molecules. First, although the polymeric bonds can be most likely formed within each fullerene layer due to the special arrangement of C_60_/Fc under pressure, the suitable temperature must be chosen because the temperature will affect the rotation of C_60_s and eventually influence the bonding between C_60_s. That may be the reason why 1D polymers can be formed under cold compression in our previous work but not in the “up” run of this work. Second, due to the gasification or movement of Fc molecules with the help of temperature and accompanying by the effect of pressure, it is possible for inter-layered C_60_s to get close enough to form bonds. Thus, we can not only obtain 1D chain-like polymers and branch-like polymers within each fullerene layer, but also the 2D polymers formed between layered fullerenes. However, under room temperature only 1D polymers can be synthesized but not higher dimensional polymers. These differences thus provide us with good models for studying the effect of spatial confinement on the polymerization of doped C_60_ materials and the resulting potential to create new polymeric structures, as well as to improve our understanding of the polymerization mechanism of confined doped fullerene.

## Conclusion

In summary, *in situ* HPHT study has been carried out on C_60_ and C_60_/Fc up to 3.2 GPa and 190 °C. Our results showed that both materials can form similar types of polymers under HPHT but with different fractions, i.e. besides the most fraction of the polymers is still 1D chain-like polymer in both cases, a larger amount of 2D polymers were synthesized from pure C_60_, while more branch-like polymers were obtained from C_60_/Fc. The polymers formed in C_60_ can be detected both during the “up” run and “down” run, while in C_60_/Fc, the polymers can only be synthesized in the “down” run. The polymerization mechanism on C_60_/Fc under HPHT was also revealed in this study. We found that the different initial lattice structures of the two materials and the confinement effect of the dopant were contributed to the differences between the two cases.

## Methods

C_60_/Fc crystals were synthesized by introducing sufficient Fc (200 mg) into a certain amount of saturated C_60_/toluene solution (3 ml). After ultrasonication we then gently added 3 ml isopropyl alcohol (IPA) into the mixture. Finally, the mixture was maintained at 10 °C for 24 h for the growth of single crystalline C_60_/Fc. The detailed method has also been described in previous studies^23, 29^.

We used a special three columned diamond anvil cell (the culet is 500 micrometer) to carry out the *in situ* HPHT experiment. For comparison, pristine C_60_ and C_60_/Fc were both loaded in one gasket. In the “up” run, the samples were firstly applied to a certain pressure and then heated to target temperatures, while in the “down” run, the samples were firstly cooled down to target temperatures and then released to the given pressures. The samples were heated by the local electric resistance wires wrapped around the tungsten carbide seats which support the diamond anvils and the temperatures were recorded by a digital thermometer. In this study, no pressure medium was used and the highest pressure and temperature was 3.2 GPa and 190 °C. HPHT Raman measurements have been carried out using a Raman spectrometer (Renishaw in Via) with a 633 nm He-Ne laser line as excitation. The light spot diameter is about 1.7 micrometer and the exposure time is 50 s. The laser power is altered between 0.04–0.05 mW (for the measurement on the recovered samples to avoid the formation of photo-polymerization) and 4–5 mW (for the measurement during the experiment).

### Data Availability

The datasets generated during and/or analysed during the current study are available from the corresponding author on reasonable request.
